# Prescription of Kampo Drugs in the Japanese Health Care Insurance Program

**DOI:** 10.1155/2013/576973

**Published:** 2013-11-30

**Authors:** Kotoe Katayama, Tetsuhiro Yoshino, Kaori Munakata, Rui Yamaguchi, Seiya Imoto, Satoru Miyano, Kenji Watanabe

**Affiliations:** ^1^Human Genome Center, Institute of Medical Science, University of Tokyo, 4-6-1 Shirokanedai, Minato-ku, Tokyo 108-8639, Japan; ^2^Center for Kampo Medicine, Keio University School of Medicine, 35 Shinano-machi, Shinjuku-ku, Tokyo 160-8582, Japan; ^3^Faculty of Environment and Information Study, Keio University, 5322 Endo, Fujisawa, Kanagawa 252-0882, Japan

## Abstract

Kampo medicine or traditional Japanese medicine has been used under Japan's National Health Insurance scheme for 46 years. Recent research has shown that more than 80% of physicians use Kampo in daily practice. However, the use of Kampo from the patient perspective has received scant attention. To assess the current use of Kampo drugs in the National Health Insurance Program, we analysed a total of 67,113,579 health care claim records, which had been collected by Japan's Ministry of Health, Labour and Welfare in 2009. We found that Kampo drugs were prescribed for 1.34% of all patients. Among these, 92.2% simultaneously received biomedical drugs. *Shakuyakukanzoto* was the most frequently prescribed Kampo drug. The usage of frequently prescribed Kampo drugs differed between the youth and the elderly, males and females, and inpatients and outpatients. Kampo medicine has been employed in a wide variety of conditions, but the prescription rate was highest for disorders associated with pregnancy, childbirth, and the puerperium (4.08%). Although the adoption of Kampo medicine by physicians is large in a variety of diseases, the prescription rate of Kampo drugs is very limited.

## 1. Introduction

Interest in traditional medicine has increased globally in recent years. The World Health Organization (WHO) recommends that physicians use traditional medicine for care and treatment and encourages the integration of traditional medicine into the next edition of the International Statistical Classification of Diseases and Related Health Problems (ICD-11) [1–4].

Kampo medicine (Japanese traditional medicine) became integrated into the Japanese health care system—the National Health Insurance Program—46 years ago alongside modern medicine, and the program has covered all citizens since 1961.

Because there is only one type of medical license in Japan, physicians prescribe both Kampo medicine and western biomedicine. At present, 148 Kampo extracts are approved as prescription drugs. Currently, 80–90% of physicians use Kampo drugs in daily practice [5–9], but most use a very limited number of Kampo drugs. In that sense, the number of patients receiving prescriptions for Kampo drugs is unknown. Similarly, their joint use with biomedical drugs has not received the appropriate amount of attention. In this study, therefore, we examine Kampo drug prescription within the Japanese national health care system.

## 2. Material and Method

The Ministry of Health, Labour and Welfare (MHLW) of Japan collects health care claims data annually. The MHLW conducts an annual survey in order to assess the medical status of treatments, diseases and injuries, and drug administration; obtain basic data for the management of medical insurance; and report on the use of drugs under the health insurance system managed by the Japan Health Insurance Association, society-managed employment-based health insurance schemes, the national health insurance scheme, and the medical care system for the elderly in later stages of life. We elucidate the usage of Kampo drugs by examining MHLW's records from 2009.

We assessed detailed statements selected from a stratified random two-stage sampling process; insurance-covered medical care institutions and pharmacies formed the primary sampling unit; and detailed statements were the secondary sampling unit (Table 1). We analysed the retrieved data for age, sex, diagnostic name, use of drugs (designation and dosage), and the number of prescriptions dispensed to inpatients and outpatients.

## 3. Results

The MHLW approves of three categories of Kampo drugs for reimbursement under the National Health Insurance Program. These include Kampo formulation extracts (extracts based on Kampo preparation formulae), crude drugs (including powdered crude drugs), and crude drug preparations. Of the 382 categories of approved drugs covered by public health insurance, they are the 28th (0.80%), 177th (0.05%), and 238th (0.02%) most-prescribed categories, respectively (numbers in parentheses indicate the quantity of Kampo drugs as a percentage of the total number of retrieved and prescribed drugs). In the following analyses, only drugs of the first category—the Kampo formulation extracts, which are largely provided as granules or powders of extracts from multiple crude drugs—were included. Hereafter, we will refer to Kampo formulation extracts as *Kampo extracts*.

Table 2 shows descriptive statistics for the usage of Kampo drugs in patients' sex, type, and age. Kampo drugs accounted for 1.34% of the total number of prescriptions (1.04% and 1.57% for male and female patients, resp.), with Kampo prescription being more frequent in female patients. As with other drugs, Kampo drugs were more frequently prescribed for outpatients (92.99%) than for inpatients (7.01%); this, however, was not the case for all Kampo extracts. Details are provided in a later section. We also found that Kampo drugs were prescribed more frequently than other drugs for young (under 20 years old) and elderly (older than 70 years old) patients.

We also assessed the joint use of Kampo extracts and biomedical drugs and found that 92.2% of patients who had been prescribed Kampo extracts were coadministered biomedical drugs during single consultations. The large percentage of patients undergoing combinatorial treatment reflects the uniqueness of the Japan's medical license and health care system, according to which physicians are allowed to prescribe both Kampo extracts and biomedical drugs. Statistics for diseases where Kampo extracts were prescribed as treatment are available in Table 3 and based on ICD-10 categories.

As shown in Table 4, the disease class “XV: pregnancy, childbirth, and the puerperium” contained the highest percentage (4.05% of the total number of patients in the class) of patients who were prescribed Kampo extracts; this may be because Kampo extracts are known to have fewer and relatively milder effects than biomedical drugs in pregnant and postpartum women. The next most frequent disease categories were “XIV: diseases of the genitourinary system” (3.08%) and “XI: diseases of the digestive system” (2.94%). Kampo extracts are, therefore, frequently prescribed to women and patients with digestive diseases. They were rarely used for diseases of the eye and adnexa (category VII; 0.09%) and congenital malformations, deformations, and chromosomal abnormalities (category XVII; 0.21%). None of the patients categorized into “XVI: certain conditions originating in the perinatal period” received prescriptions for Kampo extracts.

Table 5 shows the top ten most-prescribed Kampo extracts. *Kakkonto, tokishakuyakusan*, and *shoseiryuto* were prescribed for younger patients. *Shakuyakukanzoto*, *daikenchuto*, and *goshajinkigan, *meanwhile, were prescribed for elderly patients. *Daikenchuto* and *goshajinkigan* were more often prescribed for males than for females; *tokishakuyakusan* and *bofutsushosan*, in turn, were mostly prescribed for female patients. As with other traditional medicines, Kampo medicine is generally used as primary care for outpatients. Nevertheless, *daikenchuto* was more frequently administered to inpatients than outpatients.

The most prescribed Kampo drug was *shakuyakukanzoto*, which is widely used in musculoskeletal disorders such as muscle cramps and lumbago or associated conditions, such as dysmenorrhea. *Shakuyakukanzoto* acts on not only skeletal [10] but also smooth muscles [11].

Some Kampo extracts were specifically used for certain diseases; *bakumondoto*, for instance, was most frequently prescribed for bronchitis or bronchial asthma. Other Kampo extracts were applied to a wide variety of disease categories: *hochuekkito*, for instance, is prescribed for diseases of the circulatory system (category IX), digestive diseases (category XI), and conditions affecting the genitourinary system (category XIV), among others. Many Kampo extracts are coadministered with biomedical drugs. *Kakkonto* was prescribed with gargles and troches (13.23%), compresses containing anti-inflammatory chemicals (15.32%), or antipyretics (5.75% (acetaminophen)). *Goshajinkigan* was often used with anticancer drugs (20.24%).

## 4. Discussion

The Japanese health care system is unique in that Japan is the only country where traditional medicine is fully integrated alongside with modern medicine in daily practice. Currently, Japanese physicians, all trained in western medical schools, employ both biomedicine and traditional Japanese Kampo medicine in the clinic and university hospitals [5]. Kampo medicine, which originated in ancient China, had been Japan's primary health care system for over 1,500 years prior to the Meiji Restoration (1868–1912). In 1874, the government approved the Medical Care Law, which called for the adoption of the German model of health care and only legitimized western medical licenses. Early-twentieth-century physicians, however, continued to work towards reinstating Kampo as an official part of Japan's health care system.

In 1967, the first four Kampo extracts were approved for reimbursement under the National Health Insurance system. At the time of publication, this number has increased to 148 Kampo formulation extracts, 241 crude drugs, and 5 crude drug preparations. The present study examined only Kampo extracts because most Kampo drugs are prescribed in this form. The government also established the Good Manufacturing Practice (GMP) law in 1987 to ensure that all Kampo products are of uniformly high quality. The pharmaceutical industry, too, focused on Kampo medicine, engaging in research and development of high quality Kampo extracts. During the 52-year history of National Health Insurance in Japan, traditional medicine was also covered; as a result, both medicines coexist in one system [5].

However, the current status of traditional medicine's integration was largely unknown despite the MHLW's systematically collecting data on more than 60 million health care claims every year. We have analysed the use of Kampo medicine on the basis of these data.

We found that 1.34% of all patients were prescribed Kampo drugs. This appears small when contrasted with the fact that 70–80% of Japanese physicians prescribe Kampo drugs in their daily practice [5–9]. How can we explain this gap? Even though most physicians use Kampo drugs in daily practice, the proportion of patients who receive prescriptions for Kampo drugs is very small. Most physicians use only biomedicine and occasionally add Kampo drugs. Although we can speculate that the indications of Kampo drugs are very limited, Table 4 shows that all types of diseases are covered by Kampo treatment.

In Japan, most physicians are educated to be specialists. The Japanese government considers this to be problematic in an ageing society like Japan's. Elderly patients have multiple complaints at once, and general physicians are in demand in Japan. Our results show that indications of Kampo drugs are limited to one physician according to that physician's specialty, perhaps due to a western biomedical diagnosis. This is not in accordance with traditional use of Kampo drugs: Kampo medicine is a system similar to general medicine.

Until 2001, very few medical schools taught Kampo medicine. Most of physicians had not been educated in either general or Kampo medicine. In 2001, the Ministry of Education, Science, and Technology set new guidelines that incorporated Kampo education into the core curriculum of Japanese medical schools. Additionally, medical society features more and more the importance of general medicine. This may lead to a change in physicians' attitudes in the near future.

In the present study, we found that the use of Kampo extracts is relatively common in women with category XV health conditions. This may explain that why female patients are prescribed Kampo more frequently than are male patients; menopausal syndromes [12–15] and premenstrual syndrome are good indications of Kampo medicine and exclusively affect women.

The most frequently prescribed Kampo extract was *shakuyakukanzoto*, which is indicated for muscle cramp, lumbago, and abdominal pain. This drug is used as a muscle relaxant. Because it is so effective, many physicians use it as an alternative to analgesics. This Kampo extract is composed of 6 grams of peony root and 6 grams of liquorice root. Liquorice root contains glycyrrhizin and may cause pseudoaldosteronism, which can be very severe in rhabdomyolysis. Interestingly, liquorice root is included in 70% of the aforementioned 148 Kampo extracts. Physicians sometimes combine Kampo extracts, which increases the risk of pseudoaldosteronism [16]. To avoid this, proper education and drug information are necessary.

The use of Kampo has been modernized with western biomedicine. For example, the most commonly used Kampo drug for digestive diseases was *daikenchuto*. In *Jinkui Yaolue*, an ancient textbook of the second century, *daikenchuto,* was indicated for abdominal pain caused by the cold. Currently in Japan, *daikenchuto* is used for prevention of postsurgical ileus. Yasunaga et al. also showed that *daikenchuto* improved postoperative ileus requiring long-tube decompression [17]. Because the mechanism of action is well understood [18–20], many surgeons use *daikenchuto* routinely after operation; some hospitals have even adopted *daikenchuto* into their established clinical course. This is why *daikenchuto* is used more frequently in hospital settings than other Kampo extracts, which are generally used for outpatients in primary care settings.

Our data shows that 92.2% of patients prescribed Kampo extracts used them in combination with biomedical drugs. *Goshajinkigan* is one such Kampo extract that is often prescribed with anticancer drugs. It is frequently used to reduce numbness of the extremities, an adverse effect of anticancer drugs [21].

Another example is *kakkonto*, which is mainly prescribed for the treatment of the common cold. It was coadministered with gargles and troches. We also found that it was prescribed with antipyretics at a considerably high rate. In the view of Kampo medicine, such a combination is not ideal: one of the main effects of *kakkonto* is to raise the body temperature in order to eliminate high-temperature-sensitive viruses. If antipyretics are used in combination with *kakkonto*, the effect of *kakkonto* will be masked. It is possible that such misuse occurs because physicians ignored Kampo medicine theory and prescribed it in the light of westernized biomedicine.

There are only 2,420 board-certified Kampo doctors among the 280,000 licensed physicians of Japan. Historically, Kampo treatment was advised based on the patient's total body pattern. For effective Kampo treatment, drugs must be used in their traditional way. Additionally, although many physicians demand clinical evidence of Kampo medicine, most of the Kampo clinical studies have been performed based on western diagnoses [22].

Therefore, in order to prescribe Kampo extracts properly and avoid their misuse, education and clinical evidence based on traditional use of Kampo medicine should be established. In order to do so, standardized Kampo diagnosis is necessary. The WHO sets out to create an international classification of traditional medicine, and plans to incorporate it into the ICD-11 revision [2–4]. Mutual communication between traditional medicine and biomedicine specialists will thereupon be promoted and become more visible internationally. It is necessary to extend Kampo education in medical schools and to set up systematic continuous Kampo education after graduation. The safety and effectiveness of Kampo treatments are of utmost importance. Knowledge of Kampo diagnosis and proper use of Kampo drugs are, therefore, essential. This will ensure that future generations of Japanese physicians are able to truly integrate Kampo medicine into their practice.

## 5. Conclusion

We shed light on the prescription rate of Kampo drugs in Japan, analysing 67,113,579 health care claims records collected by the Ministry of Health, Labour and Welfare. The rate of Kampo prescription is still very low compared to the high rates of physicians' use of Kampo drugs. This gap may be caused by the Kampo drugs that were not used based on Kampo medicine theory, but by western biomedical diagnosis. Kampo drugs are more effective when they are used in the proper Kampo way, and proper education in this regard may be necessary.

## Figures and Tables

**Table 1:** Numbers of institutions and detailed statements reviewed in the surveillance study.

	Number of medical institutions	Number of detailed statements		
		Total	General medical care	Medical care system for the elderly in the later stages of life
Medical institutions	11,133	359,489	221,272	138,217
Hospitals	1,409	115,447	78,299	37,148
Clinics	9,724	244,042	142,973	101,069
Dental care institutions	1,004	28,391	18,175	10,216
Pharmacies	4,816	77,121	47,037	30,084

**Table 2:** Distributions of the usage of Kampo drugs for sex, patient type, and age.

Drug	Total^1^	Sex^2^		Patient		Age									
		Male	Female	Inpatient	Outpatient	0–9	10–19	20–29	30–39	40–49	50–59	60–69	70–79	80–89	90–98
All	67,095,008	42.81%	57.19%	2.88%	97.12%	9.05%	4.70%	5.28%	7.86%	7.82%	11.19%	18.58%	21.75%	11.82%	1.95%
Kampo	898,797	33.09%	66.91%	7.01%	92.99%	1.57%	1.76%	6.89%	9.80%	10.69%	9.85%	17.55%	23.81%	15.49%	2.59%

^1^Kampo drugs were perscribed for 100(89,897/67,095,0081) = 1.34% patients of the total number of prescription.

^2^For male, Kampo drugs were prescribed for 100((89,897 × 0.3309)/(67,095,008 × 0.4281)) = 1.04% patients. For female 100((89,897 × 0.6691)/(67,095,008 × 0.5719)) = 1.57%.

**Table 3:** Disease classification based on the International Classification of Diseases (ICD) 10th revision for 2008.

Category	Description
I	Certain infectious and parasitic diseases
II	Neoplasms
III	Diseases of the blood and blood-forming organs and certain disorders involving the immune mechanism
IV	Endocrine, nutritional, and metabolic diseases
V	Mental and behavioural disorders
VI	Diseases of the nervous system
VII	Diseases of the eye and adnexa
VIII	Diseases of the ear and mastoid process
IX	Diseases of the circulatory system
X	Diseases of the respiratory system
XI	Diseases of the digestive system
XII	Diseases of the skin and subcutaneous tissue
XIII	Diseases of the musculoskeletal system and connective tissue
XIV	Diseases of the genitourinary system
XV	Pregnancy, childbirth, and the puerperium
XVI	Certain conditions originating in the perinatal period
XVII	Congenital malformations, deformations, and chromosomal abnormalities
XVIII	Symptoms, signs, and abnormal clinical and laboratory findings, not elsewhere classified
XIX	Injury, poisoning, and certain other consequences of external causes

http://apps.who.int/classifications/icd10/browse/2008/en.

**Table 4:** Ratios of Kampo extract use per ICD-10 category.

ICD-10 category	Fraction of patients prescribed Kampo drugs per ICD-10 category		Fraction of patients per ICD-10 category		Ratio of patients prescribed Kampo drugs to total number of patients
I	37,458	(4.17%)	2,698,197	(4.02%)	1.39%
II	34,688	(3.86%)	2,811,687	(4.19%)	1.23%
III	2,933	(0.33%)	243,543	(0.36%)	1.20%
IV	81,481	(9.06%)	6,020,386	(8.97%)	1.35%
V	50,770	(5.65%)	3,171,006	(4.72%)	1.60%
VI	23,817	(2.65%)	1,752,559	(2.61%)	1.36%
VII	5,836	(0.65%)	6,865,056	(10.23%)	0.09%
VIII	3,671	(0.41%)	1,142,209	(1.70%)	0.32%
IX	189,799	(21.11%)	13,456,856	(20.05%)	1.41%
X	117,320	(13.05%)	8,920,202	(13.29%)	1.32%
XI	110,688	(12.31%)	3,761,886	(5.61%)	2.94%
XII	36,727	(4.08%)	4,178,405	(6.23%)	0.88%
XIII	101,762	(11.32%)	6,336,119	(9.44%)	1.61%
XIV	72,101	(8.02%)	2,344,046	(3.49%)	3.08%
XV	10,501	(1.17%)	259,084	(0.39%)	4.05%
XVI	0	(0.00%)	55,404	(0.08%)	0.00%
XVII	336	(0.04%)	161,658	(0.24%)	0.21%
XVIII	7,459	(0.83%)	777,263	(1.16%)	0.96%
XIX	11,835	(1.32%)	2,158,013	(3.22%)	0.55%

Total	899,182	(100.00%)	67,113,579	(100.00%)	1.34%

**Table 5:** The ten most-frequently used Kampo extracts and their use rates according to age, sex, hospitalization, and ICD-10 categories.

Rank	1	2	3	4	5	6	7	8	9	10
	Shakuyakukanzoto extracts	Kakkonto extracts	Daikenchuto extracts	Hochuekkito extracts	Shoseiryuto extracts	Tokishakuyakusan extracts	Rikkunshito extracts	Bofutsushosan extracts	Bakumondoto extracts	Goshajinkigan extracts
Total	79,580	72,224	45,090	37,058	31,625	27,941	26,513	25,784	24,545	23,253
Age^1^	71.73	54.84	70.16	62.36	53.83	48.40	63.11	56.42	55.86	72.65
Male	31.61%	31.13%	52.30%	34.50%	34.41%	1.66%	33.67%	8.90%	32.09%	47.68%
Female	68.39%	68.87%	47.70%	65.50%	65.59%	98.34%	66.33%	91.10%	67.91%	52.32%
Inpatients	4.63%	2.47%	38.59%	7.50%	3.69%	4.14%	12.26%	3.45%	3.67%	7.79%
Outpatients	95.37%	97.53%	61.41%	92.50%	96.31%	95.86%	87.74%	96.55%	96.33%	92.21%
I	1.34%	3.08%	2.29%	1.40%	1.15%	7.89%	2.26%	0.33%	0.02%	3.32%
II	3.84%	0.80%	17.00%	5.49%	1.58%	2.05%	5.28%	0.04%	1.12%	20.24%
III	0%	0.07%	0.14%	0%	0.07%	0.05%	0%	0%	0.24%	0%
IV	10.54%	6.33%	3.31%	8.88%	3.11%	14.41%	6.05%	15.44%	6.25%	10.08%
V	1.12%	2.23%	12.95%	9.05%	2.94%	1.90%	8.18%	6.36%	0.00%	1.86%
VI	0.46%	4.13%	6.18%	4.52%	0.40%	5.38%	6.48%	1.65%	2.63%	3.17%
VII	0%	0.35%	0%	1.40%	1.93%	0%	0%	0.00%	1.31%	2.15%
VIII	0%	0.14%	0%	0%	0%	0%	0%	0.00%	0.00%	10.15%
IX	27.88%	26.58%	17.48%	23.89%	16.94%	11.53%	16.28%	33.05%	9.61%	13.64%
X	3.68%	26.66%	5.40%	5.96%	27.42%	1.88%	10.69%	0.13%	59.15%	4.29%
XI	5.34%	8.31%	25.70%	11.38%	24.53%	8.05%	32.60%	8.44%	5.36%	4.04%
XII	1.19%	0.58%	0.82%	7.73%	1.22%	1.35%	0.04%	15.38%	0%	0.08%
XIII	34.89%	13.38%	1.61%	7.16%	5.57%	8.68%	6.79%	13.83%	6.66%	19.39%
XIV	5.32%	4.97%	1.10%	10.47%	10.54%	30.71%	3.91%	3.63%	4.47%	6.46%
XV	0.04%	2.07%	0.48%	0.15%	1.21%	4.97%	0.06%	0.00%	2.62%	0%
XVI	0%	0%	0%	0%	0%	0%	0%	0%	0%	0%
XVII	0%	0%	0.03%	0%	0%	0%	0.92%	0%	0%	0%
XVIII	1.07%	0.01%	2.17%	0%	0.64%	0.68%	0.45%	0.78%	0%	0.65%
XIX	3.28%	0.32%	3.34%	2.51%	0.74%	0.47%	0.02%	0.95%	0.56%	0.47%

^1^Age: the average age of the patients who were prescribed the indicated Kampo drug.
